# Novel Pharmacological and Nanotechnology-Based Therapeutic Strategies for MASLD

**DOI:** 10.3390/pharmaceutics18050584

**Published:** 2026-05-09

**Authors:** Elda Cristina Villaseñor-Tapia, Adriana Franco-Acevedo, Rebeca Rosas-Campos, Juan Armendariz-Borunda

**Affiliations:** 1 EMCS, Tecnologico de Monterrey, Zapopan 45201, Jalisco, Mexico; eldavillasenor@tec.mx (E.C.V.-T.); adrianafa@tec.mx (A.F.-A.); rosas.rc@tec.mx (R.R.-C.); 2 Department of Molecular Biology and Genomics, Institute for Molecular Biology in Medicine and Gene Therapy, University of Guadalajara, CUCS, Guadalajara 44340, Jalisco, Mexico

**Keywords:** MASLD/NAFLD, MASH/NASH, liver, nanoparticles/nanotherapies, nanomedicine, drug delivery systems, pharmacological therapies, personalized medicine

## Abstract

Metabolic dysfunction-associated steatotic liver disease (MASLD) is an increasingly prevalent global health concern driven by metabolic imbalance and excess caloric intake, leading to hepatic steatosis, inflammation, and fibrosis that may progress to metabolic dysfunction-associated steatohepatitis (MASH), cirrhosis, or hepatocellular carcinoma. Current management relies primarily on lifestyle interventions and, in advanced stages, pharmacological therapies; however, long-term outcomes remain limited due to variable efficacy and poor sustainability. Recent advances in pharmacotherapy, including GLP-1 receptor agonists, THR-β agonists and SGLT2 inhibitors, have shown clinically meaningful improvements in metabolic parameters and hepatic steatosis, although their impact on fibrosis and long-term disease modification remains uncertain. In parallel, genomic and nanotechnology-based strategies (such as RNA-based therapies and nanoparticle delivery systems) have emerged as promising approaches to enhance drug stability, targeting, and therapeutic precision. Despite these advances, most emerging strategies remain at preclinical or early translational stages, with significant challenges related to safety, scalability, regulatory approval, and long-term efficacy. In this context, this review provides an integrative synthesis of pharmacological, genomic, and nanotechnology-based therapies, highlighting their mechanisms, limitations, and translational potential. Future research should focus on well-designed clinical trials, standardized evaluation frameworks, and the development of personalized therapeutic approaches. The convergence of these strategies may enable more effective and durable interventions for MASLD.

## 1. Introduction

The terminology of non-alcoholic fatty liver disease (NAFLD) has recently been revised to metabolic dysfunction-associated steatotic liver disease (MASLD). This condition impacts nearly 40% of the adult population worldwide [1]. MASLD is distinguished by excessive hepatic fat accumulation (≥5% of hepatocytes), which may progress to metabolic dysfunction-associated steatohepatitis (MASH), the severe phenotype of the disease. Previously referred to as non-alcoholic steatohepatitis (NASH), MASH is histologically defined by lobular inflammation, hepatocyte ballooning, and fibrosis. If not reversed, progressive liver injury may culminate in cirrhosis and hepatocellular carcinoma (HCC) [2,3].

The pathogenesis of MASLD is multifactorial, involving intricate interactions among metabolic, genetic, and environmental factors. The disease typically begins with excessive hepatic lipid accumulation, resulting from an imbalance between lipid uptake, synthesis, oxidation, and export. Elevated free fatty acids increased de novo lipogenesis, and dietary lipid overload collectively promote hepatocellular triglyceride deposition, leading to hepatocyte injury and apoptosis [4]. Damaged hepatocytes release damage-associated molecular patterns (DAMPs), which activate Kupffer cells and hepatic stellate cells (HSCs), inducing the secretion of pro-inflammatory cytokines (TNF-α, IL-1β, IL-6) and fibrogenic mediators (TGF-β, PDGF). These events amplify inflammation, enhance extracellular matrix (ECM) deposition, and impair β-oxidation, ultimately culminating in fibrosis and architectural remodeling of the liver parenchyma [4,5]. The predominant risk factors for MASLD include excessive caloric intake and physical inactivity, although genetic predisposition, alcohol consumption, smoking, and coexisting metabolic disorders also contribute to disease onset and progression [6].

Liver biopsy remains the gold standard for diagnosing steatohepatitis and fibrosis; however, its invasive nature limits routine clinical use. Non-invasive alternatives include serum and imaging biomarkers. Serum parameters typically evaluated are fasting glucose, insulin, lipid profiles, and, in some cases, transaminase levels. Additional indicators such as hemoglobin A1c, blood pressure, waist circumference, body mass index (BMI), and body fat percentage provide complementary diagnostic value [7]. Imaging modalities include vibration-controlled transient elastography, shear wave elastography, acoustic radiation force impulse imaging, and magnetic resonance elastography [8,9]. Early-stage MASLD represents a critical therapeutic window, as metabolic alterations and hepatic steatosis remain potentially reversible, highlighting the importance of early detection and intervention strategies.

**Figure 1:**
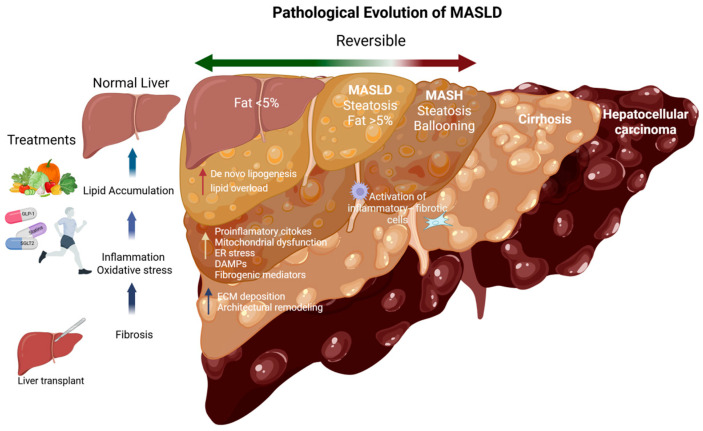
Pathological evolution of MASLD. The disease continuum progresses from normal liver to simple steatosis (MASLD), and metabolic dysfunction-associated steatotic liver disease (MASH), which is characterized by steatosis, inflammation, hepatocyte ballooning, and fibrosis. If left untreated, the disease may progress to cirrhosis and ultimately HCC (hepatocellular carcinoma). Early stages are potentially reversible through lifestyle modifications (dietary interventions, physical activity) and pharmacological approaches, whereas advanced stages are generally irreversible and may require liver transplantation.

In the early stages of MASLD, lifestyle modification, dietary restriction, and structured physical activity remain the cornerstone of therapy. In advanced disease, bariatric or metabolic surgery may be considered [3]. Pharmacological options currently regarded as optimal include thyroid hormone receptor agonists (e.g., *Resmetirom*), glucagon-like peptide-1 (GLP-1) receptor agonists (e.g., *Liraglutide*), sodium–glucose cotransporter 2 (SGLT2) inhibitors, statins, and metformin [3]. Ongoing or actively recruiting clinical trials are investigating fibroblast growth factor 21 (FGF21) analogues, such as *Efruxifermin* and *Pegozafermin* [3,10,11] (Figure 1).

Despite these therapeutic options, the effectiveness of current management strategies remains limited, primarily due to poor long-term adherence to lifestyle modifications. Consequently, there is an urgent need to identify and develop innovative therapeutic approaches capable of providing sustained clinical benefits [12]. Given the multifactorial nature of MASLD, therapeutic interventions must target multiple molecular pathways simultaneously rather than isolated pathogenic processes [13].

This review aims to critically evaluate emerging pharmacological, genomic, and biotechnological approaches for the treatment of MASLD, with the goal of advancing durable and effective therapies. Although MASLD encompasses a continuum of pathological stages from simple MASH to cirrhosis and HCC, the present work focuses on therapeutic and drug delivery strategies that target shared molecular mechanisms underlying disease progression (Figure 1).

To enhance conceptual integration, we propose a classification framework that stratifies MASLD therapeutic strategies into three levels: (i) systemic metabolic modulators (e.g., GLP-1RAs, SGLT2 inhibitors), (ii) targeted molecular therapies (e.g., FGF21 analogs, THR-β agonists), and (iii) precision nanomedicine and gene-based interventions. This framework further distinguishes approaches based on translational maturity (clinical vs. preclinical), enabling a clearer comparison of therapeutic potential and limitations.

## 2. Search Strategy and Scope

This work was conducted as a narrative review aimed at providing a conceptual and integrative synthesis of emerging pharmacological, genomic, and nanotechnology-based therapeutic strategies for MASLD.

A structured literature search strategy was employed to ensure comprehensive coverage, as its objective is not quantitative synthesis but critical and translational interpretation of heterogeneous evidence. The literature search included PubMed, Scopus, Web of Science, Embase, and ClinicalTrials.gov (January 2020–present).

Search terms were grouped into three categories: (i) disease-related (MASLD, MASH, NAFLD, NASH), (ii) therapeutic strategies (pharmacological agents, natural compounds), and (iii) advanced technologies (nanoparticles, gene therapy, mRNA, CRISPR).

Therapeutic strategies were further classified according to level of evidence (clinical vs. preclinical) and translational maturity.

## 3. Novel Pharmacological Agents

**Figure 2:**
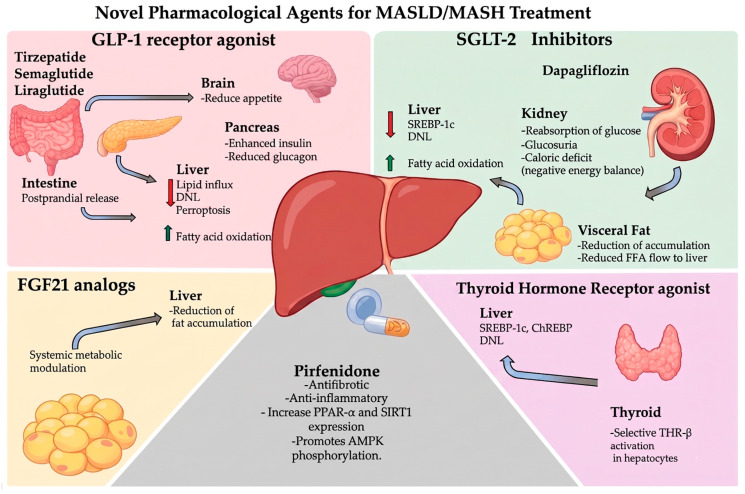
Pharmacological strategies targeting metabolic pathways in MASLD/MASH. merging drug treatments for MASLD and MASH. GLP-1 receptor agonists improve metabolism by suppressing appetite centrally, increasing pancreatic insulin secretion, decreasing glucagon release, and reducing hepatic lipid influx and de novo lipogenesis (DNL). SGLT-2 inhibitors block renal glucose reabsorption, leading to glucosuria and a negative energy balance that helps reduce visceral fat and the flow of free fatty acids to the liver, thus decreasing hepatic lipogenesis and increasing fatty-acid oxidation. FGF21 analogs have systemic metabolic effects that lower hepatic lipid buildup and enhance overall energy metabolism. Thyroid hormone receptor-β agonists activate liver-specific thyroid hormone signaling, suppressing lipogenic transcription factors such as SREBP-1c and ChREBP, thereby reducing DNL. Pirfenidone, with antifibrotic and anti-inflammatory effects, up-regulates PPAR-α and SIRT1 and promotes AMPK phosphorylation, thereby improving hepatic lipid metabolism and reducing fibrosis. These treatments target various organs and pathways to slow or reverse the progression of MASLD and MASH.

MASLD treatment is primarily focused on lifestyle and behavioral changes. However, once fibrosis is present, therapeutic approaches become more complex [13]. Recently, new therapeutic alternatives with pharmacological, genomic, and biotechnology-based strategies have emerged to slow disease progression [13] (Figure 2).

Recently, the Food and Drug Administration approved a GLP-1 receptor agonist as a treatment for MASDL [14]. Tirzepatide has been documented as a treatment for MASLD/MASH secondary to obesity complications [15]. This decision is based on the fact that GLP-1 is an intestinal incretin that, when released postprandially, enhances glucose-dependent insulin secretion, reduces glucagon, slows gastric emptying, and decreases appetite, promoting weight loss and improved insulin resistance; two key determinants of hepatic fat accumulation [16]. In the context of MASH, it has been proposed that GLP-1 receptor agonists (GLP-1RAs) reduce lipid influx into the liver and modulate metabolic pathways involved in de novo lipogenesis (DNL) and fatty acid oxidation by activating the AMPK/ACC pathway and suppressing ferroptosis [16,17,18].

In clinical trials, histological outcomes, liraglutide (LEAN trial, 48 weeks), liraglutide achieved NASH resolution without worsening of fibrosis in 39% of patients compared with 9% in the placebo group [17]. In a phase 2 trial, semaglutide led to NASH resolution in 59% of patients, compared with 17% with placebo, although fibrosis improvement did not reach statistical significance [18].

On the other hand, the SGLT-2 is responsible for renal glucose reabsorption; its inhibition (iSGLT-2) induces glucosuria, reduces blood glucose levels, and generates a caloric deficit with weight loss, effects that may translate into improved MASH activity [19]. Although iS-GLT-2 does not act directly on hepatocytes, it lowers circulating insulin, reducing activation of insulin-dependent lipogenic factors such as SREBP-1c, thereby decreasing DNL and triglyceride accumulation in hepatocytes [20]. The caloric loss from glucosuria creates a negative energy balance, reducing visceral fat and free fatty acid flow to the liver. This promotes catabolic pathways, including AMPK activation, which boosts mitochondrial β-oxidation and inhibits lipid storage. Consequently, lipotoxic species such as diacylglycerols and ceramides decrease, preventing hepatocellular dysfunction, insulin resistance, and progression to MASH. Reduced inflammation, oxidative stress, and liver damage also lower profibrogenic signals associated with activation of these pathways in cells [13,20,21].

In a double-blind trial with dapagliflozin (48 weeks, biopsy), a reduction in histological steatosis score was observed (mean change −0.82) compared with placebo (−0.37), with a significant difference between groups [22].

Thyroid hormone receptor agonists (THR agonists) are a therapeutic strategy aimed at modulating hepatic lipid metabolism through the selective activation of the beta thyroid hormone receptor (THR-β), the predominant isoform in the liver. At the molecular level, activation of THR-β in hepatocytes induces the transcription of genes involved in the uptake, oxidation, and elimination of lipids, including those related to mitochondrial and peroxisomal β-oxidation, fatty acid transport, and cholesterol metabolism. In parallel, THR-β-mediated signaling suppresses the expression of lipogenic genes regulated by SREBP-1c and ChREBP, resulting in an inhibition of DNL. This dual effect includes increased lipid catabolism and reduces fatty acid syn-thesis, leading to a significant decrease in intrahepatic triglyceride content [23,24,25,26].

From a clinical perspective, selective THR-β agonists (Resmeritom) have demonstrated a robust reduction in hepatic steatosis evaluated by imaging techniques and histology. In advanced clinical trials with resmetirom, a selective THR-β agonist, a significant reduction in liver fat content has been observed, along with histological improvement of steatosis, defined as a decrease of at least one grade in the steatosis component of the NAFLD Activity Score (NAS) in a substantial proportion of treated patients compared to placebo. Additionally, these effects are accompanied by improvements in systemic lipid profiles, particularly a reduction in LDL cholesterol and triglycerides, reinforcing the role of THR-β as a central regulator of hepatic lipid metabolism. Also; Resmetirom has recently received FDA approval for the treatment of noncirrhotic MASH with fibrosis, representing a milestone in MASLD pharmacotherapy. Therefore, these medications could be useful in the comprehensive treatment of MASLD [26,27,28].

Pharmacological analogs of FGF21 have been evaluated in multiple phase 2 clinical trials in patients with MASLD/MASH, demonstrating consistent reductions in fat accumulation and improvements in liver parenchyma. An example is Pegbelfermin (BMS-986036), which has been tested in a phase 2a, double-blind, placebo-controlled trial in patients with biopsy-confirmed NASH over 16 weeks, showing a re-duction in liver fat content of approximately 30% in 50% of patients treated with 10 mg daily [29]. Similarly, in the case of Efruxifermin (AKR-001), it was evaluated in the Phase 2a BALANCED trial in patients with biopsy-confirmed NASH over 16 weeks, finding a reduction of less than 30% in fat accumulation in 80–90% of patients. Additionally, a reduction in fibrosis was observed in 48% of the patients [30].

Similarly, Pegozafermin (BIO89-100) was evaluated in the Phase 2 ENLIVEN trial in patients with NASH. The treatment was associated with significant reductions in liver fat content, with 60–70% of the patients. These results demonstrate their role as systemic metabolic modulators with an impact on hepatic lipotoxicity, inflammation, and the reduction in progression from MASLD to MASH; however, phase 3 studies with long-term follow-up are needed to strengthen the findings [11].

Pirfenidone is an oral antifibrotic medication approved for idiopathic pulmonary fibrosis. It works by decreasing profibrotic mediators like transforming growth factor-β (TGF-β) and reducing the production of fibrogenic mediators, collagen synthesis, and extracellular matrix buildup in fibrotic tissues. Additionally, it lowers pro-inflammatory cytokines such as tumor necrosis factor-α (TNF-α) and interleukin-1β (IL-1β), reflecting its anti-inflammatory effects seen in preclinical fibrosis models. In metabolic dysfunction-associated steatohepatitis (MASH), preclinical studies show that pirfenidone reduces liver inflammation and fibrosis by decreasing hepatic stellate cell activation, collagen deposition, and pro-inflammatory cytokines, along with diminishing oxidative stress in rodent liver injury models. Recent findings indicate that prolonged-release pirfenidone acts as an agonist for peroxisome proliferator-activated receptor-α (PPAR-α). In high-fat diet models of steatosis and MASH, it activates PPAR-α, increases SIRT1 expression, and promotes AMPK phosphorylation, which correlates with better lipid metabolism, less liver fat accumulation, reduced inflammation and fibrosis, and improved insulin sensitivity. By activating PPAR-α, pirfenidone enhances pathways that promote fatty acid oxidation and energy balance, exerting an anti-steatogenic effect in fatty liver disease models. Although its antifibrotic actions involve modulation of TGF-β and related pathways, evidence in humans with MASLD/MASH is limited. Small studies and cohort data suggest improvements in liver enzymes and non-invasive fibrosis markers, but because these are based on small sample sizes and short follow-up, larger randomized trials are necessary to confirm their efficacy and safety in MASLD/MASH [31,32]. To facilitate comparison across pharmacological strategies and improve clarity, key therapeutic agents are summarized in Table 1.

## 4. Nanoparticle, Biotechnology, and Genomic-Based Therapies in MASLD

It is noteworthy that nanotechnology-based platforms should not be regarded as standalone therapeutic approaches but rather as facilitating systems that augment the pharmacokinetic and pharmacodynamic attributes of pharmacological agents. By enhancing bioavailability, tissue specificity, and intracellular targeting, nanocarriers have the capacity to amplify the effectiveness of drugs, antioxidants, or gene therapies. This integrative perspective underscores the intersection of pharmacology and nanomedicine as a pivotal direction in MASLD treatment.

From nanotechnology, nanotherapies have advanced drug delivery systems and therapeutic strategies by enabling precise targeting, enhanced bioavailability, and reduced toxicity [38,39]. These innovations are reshaping paradigms in drug delivery and formulation. Multiple approaches have shown substantial potential to overcome the limitations of conventional treatments and may represent promising options for the management of MASLD. The primary objective of these formulations is to increase drug accumulation within hepatocytes or hepatic stellate cells while minimizing systemic exposure and off-target effects. Their improved bioavailability and controlled-release properties extend drug half-life, maintain optimal therapeutic concentrations, and reduce dosing frequency and enzymatic degradation. Furthermore, nanocarrier systems enhance molecular precision, promoting site-specific release and enabling the multifunctional co-delivery of pharmacological agents and genetic materials (such as siRNA, mRNA, or CRISPR–Cas9 constructs) to simultaneously modulate metabolic, inflammatory, and fibrotic pathways [40,41,42].

**Figure 3:**
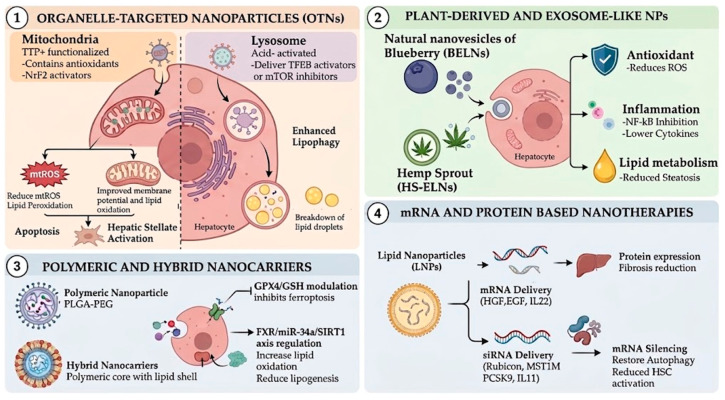
Nanotechnology-based therapeutic strategies for MASLD/MASH. Schematic overview of emerging nanomedicine approaches for the treatment of MASLD and MASH. (**1**) Organelle-targeted nanoparticles (OTNs) deliver therapies to specific cell compartments. Mitochondria-targeted systems, decorated with lipophilic cations like triphenylphosphonium (TPP^+^), transport antioxidants and Nrf2 activators to reduce mitochondrial reactive oxygen species (mtROS), lipid peroxidation, apoptosis, and hepatic stellate cell activation. Lysosome-targeted nanoparticles release acid-sensitive cargoes, such as TFEB activators or mTOR inhibitors, thereby boosting lipophagy and degrading intracellular lipid droplets. (**2**) Plant-derived nanoparticles like blueberry (BELNs) and hemp exosome-like nanoparticles (HS-ELNs) protect the liver by reducing oxidative stress, blocking NF-κB inflammation, and improving lipid metabolism, decreasing hepatic steatosis. (**3**) Polymer and hybrid nanocarriers, such as PLGA-PEG or polymer–lipid systems, offer controlled drug delivery and influence pathways like GPX4/GSH (to prevent ferroptosis) and FXR/miR-34a/SIRT1 (to promote fatty-acid oxidation and inhibit lipogenesis). (**4**) mRNA- and protein-based nanotherapies, mainly via lipid nanoparticles (LNPs), allow targeted delivery of therapeutic nucleic acids. mRNA can produce regenerative/antifibrotic proteins (e.g., HGF, EGF, IL-22), while siRNA silences disease genes (e.g., Rubicon, MST1M, PCSK9, IL-11), restoring autophagy, reducing stellate cell activation, and easing fibrosis. These nanotechnologies target key processes in MASLD/MASH progression, including oxidative stress, inflammation, lipid imbalance, and fibrogenesis.

Collectively, these nanotechnology-driven innovations represent a pivotal step toward the development of personalized, durable, and mechanism-targeted therapies for MASLD (Figure 3).

### 4.1. Organelle-Targeted Nanoparticles

Organelle-targeted nanoparticles (OTNs) are a sophisticated nanotherapeutic approach designed to improve intracellular targeting by delivering bioactive compounds specifically to certain subcellular structures [43]. Unlike traditional drug delivery methods that primarily aim at tissues or cells, OTNs are tailored to concentrate within organelles like mitochondria, lysosomes, or the nucleus. This targeted approach is especially relevant in MASLD, where organelle impairment is a key factor in disease progression [43,44].

Mitochondria-focused nanoparticles are among the most studied platforms in metabolic liver disease. They often utilize the negative potential of mitochondrial membranes by incorporating lipophilic cations such as triphenylphosphonium (TPP^+^), enabling selective mitochondrial buildup [28]. This allows for the direct delivery of antioxidants, Nrf2 activators, AMPK modulators, ferroptosis inhibitors, or gene-silencing agents to the main site of ROS production. By restoring mitochondrial redox balance and maintaining membrane potential, these particles reduce lipid peroxidation, hepatocyte apoptosis, and hepatic stellate cell activation, thereby disrupting critical fibrogenic pathways [45,46].

Nanoparticles targeting lysosomes and autophagy have also gained interest, particularly because impaired lipophagy plays a significant role in steatotic hepatocytes. These nanoparticles deliver mTOR inhibitors or TFEB activators, boosting autophagic flow and lysosomal function, which promotes lipid droplet breakdown and metabolic reprogramming. Considering the close interaction between AMPK/mTOR signaling, oxidative stress, and lipid metabolism, regulating autophagy via organelle-specific systems offers a promising therapeutic approach [47,48].

From a design perspective, effective organelle-targeted nanoparticles must overcome multiple biological barriers, including systemic circulation stability, hepatic accumulation, cellular internalization, endosomal escape, and precise organelle trafficking. Surface functionalization with targeting ligands, biodegradable polymers, redox-sensitive linkers, and pH-responsive materials enhances specificity and reduces off-target toxicity. Importantly, these platforms enable combination strategies, allowing simultaneous modulation of oxidative stress, inflammation, lipogenesis, and fibrogenesis within defined subcellular compartments [49,50].

For instance, mitochondria-targeted nanoparticles (mtTNps) attenuate high-fat diet (HFD)-induced damage and insulin resistance by regulating the Steatohepatitis-associated circRNA ATP5B Regulator (SCAR). SCAR is a mitochondria-localized circular RNA derived from the ATP5B gene, which encodes a subunit of ATP synthase and contributes to maintaining mitochondrial bioenergetics and limiting excessive production of mitochondrial reactive oxygen species. mtTNps functionalized with TPP^+^ have been engineered to restore SCAR expression or deliver nucleic acid-based modulators that enhance its activity by increasing SCAR levels. These nanocarriers reduce mtROS production, stabilize mitochondrial membrane potential, and improve oxidative phosphorylation efficiency. Preclinical studies in HFD-fed murine models have demonstrated that mitochondria-targeted nanoparticle-mediated modulation of SCAR significantly reduces hepatic steatosis, inflammatory infiltration, and fibrosis markers, while restoring glucose tolerance and insulin responsiveness. Collectively, these findings position SCAR-regulating mitochondrial nanotherapies as a highly targeted and mechanistically grounded approach for the treatment of MASLD and steatohepatitis [51]. Acid-activated biodegradable nanoparticles (acNPs) represent a novel therapeutic strategy designed to respond to the acidic microenvironment characteristic of inflamed and metabolically stressed tissues [52]. In MASLD models, these nanoparticles selectively release their therapeutic cargo under acidic conditions (such as those observed in dysfunctional lysosomes or inflamed hepatic tissue) thereby enhancing intracellular targeting efficiency while minimizing off-target effects; acNPs have been shown to significantly enhance hepatic lipid clearance by restoring autophagic flux and promoting lipophagy. This results in reduced triglyceride accumulation and attenuation of hepatocellular steatosis [48,53].

Although most organelle-targeted nanotherapies remain in preclinical stages, their mechanistic alignment with the subcellular drivers of MASLD positions them as highly promising translational strategies (Figure 3).

### 4.2. Plant-Derived and Exosome-like Nanoparticles

Plant-derived nanoparticles (PDNPs) and plant exosome-like nanoparticles (PELNs) are gaining attention as natural nanocarriers with bioactive and therapeutic potential in metabolic disorders like MASLD [54]. They resemble mammalian exosomes but differ in lipid makeup, biogenesis, and molecular cargo, giving them unique properties. PDNPs are rich in phospholipids such as phosphatidic acid, phosphatidylcholine, and phosphatidylethanolamine, along with glycolipids that enhance membrane stability and resistance to enzymes, compared to synthetic nanoparticles. Plant vesicles are more biocompatible, less immunogenic, and easier to produce from renewable sources, ideal for long-term treatment of metabolic conditions; PDNPs offer a promising new nanotherapy. Their ability to target oxidative stress, inflammation, lipid metabolism, and microbiota makes them versatile candidates in treating metabolic diseases, MASLD [54,55].

Blueberry-derived exosome-like nanoparticles (BELNs) are naturally occurring plant nanovesicles isolated from Vaccinium species that exhibit potent antioxidant and cytoprotective properties [56,57]. These vesicles, typically ranging from 50 to 300 nm in diameter, contain bioactive lipids, small RNAs, proteins, and polyphenolic compounds intrinsic to blueberries, which collectively contribute to their therapeutic potential [57]. BELNs have been shown to attenuate oxidative stress by reducing intracellular and mitochondrial reactive oxygen species (ROS) accumulation. They enhance activation of the Nrf2/Keap1 pathway. Experimental in vitro and in vivo models show that they preserve mitochondrial membrane potential, reduce cytochrome c release, and downregulate pro-apoptotic proteins such as Bax while maintaining anti-apoptotic Bcl-2 expression. Additionally, improvements in mitochondrial function and fatty acid β-oxidation capacity further reduce lipid accumulation in hepatocytes [56].

Similarly, other plant-derived nanovesicles, such as hemp sprout-derived exosome-like nanoparticles (HS-ELNs), are isolated from germinated Cannabis sativa sprouts that exhibit intrinsic bioactive and hepatoprotective properties. Preclinical studies have demonstrated that HS-ELNs exert antioxidant and anti-inflammatory effects in metabolic liver disease models [58]. These nanovesicles reduce intracellular ROS production, enhance endogenous antioxidant defenses, and modulate Nrf2/Keap1 pathway [58]. In addition, HS-ELNs attenuate hepatic inflammation by suppressing NF-κB activation and reducing expression of pro-inflammatory cytokines. Furthermore, have been associated with decreased expression of fibrotic markers, including α-SMA and collagen type I, suggesting a potential role in mitigating early fibrogenic signaling [59].

Despite all the advantages and benefits mentioned above, more research is needed to standardize isolation methods, the diversity of vessels, the quality and safety of the source and destination, as well as to expand knowledge in pharmacology, safety, effectiveness, and toxicity to ensure proper use in humans (Figure 3).

### 4.3. Polymeric and Hybrid Nanocarriers

Polymeric nanoparticles are typically constructed from biodegradable and biocompatible materials such as poly (lactic-co-glycolic acid) (PLGA), polyethylene glycol (PEG), chitosan, polycaprolactone (PCL), and other FDA-approved polymers [60]. Their architecture permits encapsulation of hydrophobic and hydrophilic molecules, nucleic acids, peptides, or small interfering RNAs (siRNAs), protecting them from premature degradation and enhancing systemic circulation time. Surface modification strategies, including PEGylation or ligand conjugation, further enhance stability and reduce opsonization and clearance by the reticuloendothelial system [61].

Hybrid nanocarriers integrate the advantages of multiple materials, combining polymeric cores with lipid shells or inorganic components such as gold nanoparticles, iron oxide, or silica matrices. These composite systems improve structural stability and enable multimodal functionality, including imaging, magnetic targeting, and stimulus-responsive drug release. Lipid–polymer hybrid nanoparticles, for example, merge the structural integrity of polymeric matrices with the biocompatibility and membrane-mimetic properties of lipid bilayers, facilitating enhanced cellular uptake and endosomal escape [62,63,64,65].

Overall, polymeric and hybrid nanocarriers provide a highly versatile and customizable platform for addressing multiple pathogenic mechanisms underlying MASLD. By integrating controlled drug delivery, tissue-specific targeting, and multifunctional design, these systems represent a promising frontier in nanotherapeutic strategies for metabolic liver disease. Novel nanodelivery systems have also been engineered. A polymer composed of 18-β-glycyrrhetinic acid–chitosan oligosaccharide–N-acetylcysteine, combined with curcumin, targeted the GPX4/GSH pathway [66]. In vitro, this treatment reduced lipid peroxidation, ferroptosis, and oxidative injury. In HFD-fed mice, nanoparticle administration improved hepatic steatosis and attenuated histopathological damage, including hepatocellular ballooning and inflammatory infiltration. Importantly, ferroptosis-related proteins were normalized, supporting the central role of iron-dependent lipid peroxidation in NAFLD progression [67].

Oligochitosan-derived nanovesicles are designed to co-encapsulate an FXR agonist and a miR-34a antagomir, alleviating lipid deposition and promoting liver regeneration via the FXR/miR-34a/SIRT1 regulatory axis [68]. In vitro experiments and in vivo studies in HFD-induced MASLD mouse models demonstrated that treatment with nanovesicles downregulates miR-34s expression and significantly increases hepatic SIRT1 levels. This molecular reprogramming was associated with activation of AMPK, suppression of lipogenic transcription factors such as SREBP-1c and FASN, and enhancement of fatty acid β-oxidation pathways. Nanovesicle administration attenuated hepatic steatosis, reduced serum transaminase levels. Additionally, improvements in insulin sensitivity and inflammatory markers were observed [69]. Similarly, an oligochitosan–ursodeoxycholic acid nanocarrier encapsulating exenatide restored hepatic function in NAFLD models. These nanovesicles respond to stimuli from the hepatic microenvironment and allow controlled release of both compounds, enhancing their metabolic effects. The proposed mechanism involved activation of the SIRT1 pathway, regulating processes related to β-oxidation, lipid metabolism, and oxidative stress. The nanoencapsulated co-administration demonstrated greater efficacy compared to individual treatments, suggesting that targeted modulation of SIRT1 through combined nanotransport is a promising strategy for multifactorial therapies in NAFLD [70].

Also, Curcumin nanocomplex were evaluated in NASH model induced in hamsters infected with Opisthorchis viverrini, a model that reproduces chronic liver inflammation and fibrotic progression. The nanoformulation improved the bioavailability of curcumin and allowed greater hepatic accumulation of the active compound. Treated animals showed significant reductions in steatosis, inflammatory infiltration, and histological damage, as well as decreased serum markers of liver injury. Additionally, attenuation of oxidative stress and modulation of pro-inflammatory cytokines were observed, suggesting a hepatoprotective effect mediated by the regulation of inflammatory and antioxidant pathways. These findings support the use of nanostructured systems to optimize bioactive compounds with low solubility, such as curcumin, in the treatment of NASH associated with chronic inflammation [71].

### 4.4. Nanoparticle-Encapsulated Pharmacological Agents

Nanoparticles that encapsulate pioglitazone and vitamin E within platelet–neutrophil hybrid membranes have shown anti-inflammatory activity against NASH [72]. Celastrol-based nanoparticles decreased inflammatory signaling, lipogenesis, and the expression of lipid transporter genes, while improving insulin sensitivity and lipolysis [73,74]. Albumin-stabilized ginsenoside compound K (CK) nanoparticles promote the clearance of lipid droplets and protect against steatosis and fibrosis [73]. Hyaluronic acid–bilirubin nanoparticles inhibit hepatic stellate cell activation, proliferation, and collagen production, thereby reducing fibrosis in NASH [74]. Other innovative methods include rimonabant-encapsulated nanoparticles designed to avoid neuropsychiatric side effects; these improved insulin sensitivity and decreased liver damage in HFD models [75]. Resveratrol-loaded nanoparticles, including copper-based nanobubbles with nucleic acid aptamers, reduced inflammatory injury [76,77]. Tat-Beclin nanoparticles restored autophagy, effectively decreasing hepatic steatosis [78]. Oral honey vesicle-like nanoparticles (H-VLNs) lessened hepatic inflammation and fibrosis by modulating inflammatory pathways [79]. Platensimycin (PTM) in liposome-based nanoformulations reduced weight gain and serum lipid levels thereby preventing NAFLD development by blocking FASN and proteins involved in de novo lipogenesis [80].

Other formulations include luteolin–zinc oxide nanoparticles, which modulate the PI3K/AKT/FOXO1 [81]. Native GLP-1 nanocarriers help maintain glucose–insulin balance and delay NAFLD progression [82]. Inorganic nanoparticles enhance lipid hydrolysis and target the Nrf2/ARE-Ces2 h pathway to reduce steatosis [83]. Cyclosporine A-loaded glucosamine nanoparticles restore mitochondrial autophagy and maintain lipid–glucose balance [84]. Nicotinamide–chitosan nanoparticles reduce transaminases, lipid levels, and oxidative injury [85]. Natural cell membrane-based nanoparticles that encapsulate rosuvastatin or capsaicin also decrease hepatic lipid accumulation [86,87]. Celastrol–hyaluronic acid nanoparticles suppress macrophage activation and improve metabolic health by modulating the CD 36/PPAR pathway [88].

### 4.5. mRNA- and Protein-Based Nanotherapies

Gene-based therapeutic strategies in MASLD encompass distinct approaches with different mechanisms and translational implications. siRNA-based therapies enable transient gene silencing without altering the genome, mRNA-based delivery allows controlled expression of therapeutic proteins, whereas CRISPR/Cas9 systems enable permanent genome editing. These differences are critical in terms of durability, safety profile, and clinical applicability, with most approaches still remaining at preclinical or early translational stages.

mRNA- and protein-based nanotherapies represent pioneering tools in precision medicine, facilitating transient, controlled expression of therapeutic proteins or the direct delivery of functional biomolecules into cells. For MASLD, these platforms offer a means to modulate disease-related pathways at both the gene expression level and post-protein synthesis, without inducing permanent genomic alterations [89].

Gene- and mRNA-based nano-therapies have demonstrated potential. Lipid nanoparticles encoding hepatocyte growth factor (HGF) and epidermal growth factor (EGF) promoted hepatic proliferation and regeneration [90]. Non-mitogenic fibroblast growth factor delivered by lipid nanoparticles reversed steatosis and improved metabolic parameters [91]. Bilirubin nanoparticles regulated ceramide production by suppressing Sgpl1 and Degs1, thereby improving the metabolic environment [42].

CRISPR–Cas9-loaded nanoparticles targeting Rubicon (RUBCN), which is a key negative regulator of autophagy that inhibits autophagosome maturation by binding to Rab7 and blocking the PI3KC3-C complex. The administration of this treatment shows a reduction in expression of RUBCN, this effect was associated with an improvement in hepatic steatosis, a decrease in lipid accumulation, and attenuation of histological damage. The intervention modulated CD36 expression, a key fatty acid transporter, and restored alterations in glycerophospholipid metabolism, thereby contributing to the normalization of the hepatic lipid profile and steatosis [92]. RNA interference nanoliposomes targeting Rubicon improved autophagy and reduced mitochondrial stress [93]. Mannose-modified lipid particles delivering HMGB1-siRNA reduced hepatic inflammation and steatosis [94]. Lipid nanoparticles encoding IL-22 regulated glucose and lipid metabolism, reducing fibrosis [95]. Inhibition of PCSK9 by triantennary N-acetylgalactosamine nanoparticles mitigated lipid accumulation and hepatocellular injury [96]. Redox-unlockable Hep@PGEA nanoparticles targeting MST1 restored AMPK/SREBP-1c balance [97]. Induction of heme oxygenase-1 by nanoparticle formulations reduced insulin resistance, inflammation, and steatohepatitis in obese mice [98]. Similarly, nanoparticles delivering RNA interference against IL11 and IL11ra1 were used to selectively target hepatic stellate cells, reducing their activation, extracellular matrix deposition, and fibrosis progression in a mouse model [99]. Due to the complexity and diversity of nanotechnology-based approaches, these strategies are summarized in Table 2, including their design rationale, experimental validation, and translational limitations.

## 5. Translational Challenges and Future Perspectives

Despite substantial advances in understanding MASLD pathophysiology, the translation of emerging therapies into clinical practice remains limited. Current management continues to rely predominantly on lifestyle interventions, which frequently fail to achieve sustained metabolic and histological remission [3,5,13].

While several pharmacological agents have demonstrated clinically meaningful effects, particularly in metabolic regulation and hepatic steatosis, their impact on fibrosis resolution and long-term outcomes remains variable [11,27,37]. Moreover, heterogeneity in trial design, patient selection, and endpoint definitions limits cross-study comparability and complicates interpretation of therapeutic efficacy [13,22,29].

For nanotechnology- and gene-based strategies, the translational gap is even more pronounced. Most evidence derives from in vitro and preclinical models that do not fully recapitulate the metabolic, inflammatory, and fibrotic complexity of human MASLD.

Critical barriers include suboptimal pharmacokinetics, incomplete tissue specificity, potential immunogenicity, and insufficient characterization of long-term biodistribution and toxicity [41,49,50].

In addition, the clinical implementation of nanomedicine is constrained by manufacturing complexity, scalability limitations, and the lack of standardized regulatory frameworks for multifunctional and hybrid delivery systems [39,50,63]. These challenges are further compounded by interspecies differences that hinder extrapolation from animal models to human disease [41,49].

Finally, the marked heterogeneity of MASLD, influenced by genetic background, metabolic status, and environmental factors, underscores the need for patient stratification strategies [1,2,3].

Biomarker-driven approaches and integration of multi-omics data will be essential to improve therapeutic targeting, predict treatment response, and reduce variability in clinical outcomes [5,7].

## 6. Conclusions

This review provides an integrative overview of pharmacological, genomic, and nanotechnology-based therapeutic strategies for MASLD, highlighting their complementary roles in targeting key pathogenic mechanisms, including lipid dysregulation, inflammation, and fibrogenesis.

Although several therapeutic classes have demonstrated promising results, particularly in early clinical trials, most emerging strategies remain at limited stages of translational development. The lack of standardized evaluation criteria, insufficient long-term clinical data, and persistent safety concerns represent major obstacles to clinical adoption.

Future progress in MASLD therapy will depend on the development of robust and well-controlled clinical studies, alongside standardized frameworks for evaluating efficacy, safety, and reproducibility, particularly for nanomedicine-based platforms. In parallel, advances in molecular profiling and systems biology are expected to enable more precise patient stratification and facilitate the transition toward personalized hepatology.

Importantly, the convergence of pharmacological modulation, gene-targeted therapies, and advanced delivery systems offers a promising avenue for addressing the multifactorial nature of MASLD. Rather than single-target interventions, future therapeutic strategies will likely require integrated approaches capable of simultaneously modulating metabolic, inflammatory, and fibrotic pathways.

Collectively, these developments have the potential to shift MASLD management from symptomatic control toward mechanism-based and potentially disease-modifying interventions, with the goal of achieving sustained metabolic recovery and improved long-term clinical outcomes.

## Figures and Tables

**Table 1:** Pharmacological therapies for MASLD: mechanisms of action, level of evidence, and clinical limitations.

Therapeutic Strategy	Main Therapeutic Rationale	Level of Evidence	Main Limitations	References
**GLP-1 receptor agonists** **(Semaglutide)**	Weight loss and systemic metabolic improvement associated with histological benefit in MASH.	Phase 3 randomized evidence. ESSENCE reported improvement in liver histology in MASH with moderate-to-advanced fibrosis; FDA granted accelerated approval for Wegovy in MASH with F2–F3 fibrosis.	Accelerated approval; clinical benefit requires longer confirmation; gastrointestinal adverse effects and dependence on weight-loss response remain relevant.	[33,34] NCT04822181.
**SGLT2 inhibitors** **(Dapagliflozin)**	Improved glycemic control and energy balance, with reported histological improvement in biopsy-confirmed MASH.	Randomized placebo-controlled clinical evidence from a 2025 multicenter trial.	Not approved specifically for MASH; evidence requires replication in larger and more diverse phase 3 studies.	[22] NCT03723252.
**THR-β agonist** **(Resmetirom/Rezdiffra)**	Liver-directed reduction in hepatic fat accumulation through THR-β activation.	Phase 3 randomized evidence; FDA accelerated approval for noncirrhotic NASH/MASH with F2–F3 fibrosis.	Long-term clinical outcomes and real-world durability require confirmation; indicated population should be respected.	[27,35] NCT03900429.
**FGF21 analog** **(Pegozafermin)**	FGF21-mediated improvement in lipid metabolism, insulin sensitivity and fibrosis-related outcomes.	Phase 2b randomized clinical trial.	Not approved; phase 3 validation and long-term safety data are needed.	[11,36] NCT04929483.
**FGF21 analog** **(Efruxifermin)**	FGF21 agonism associated with NASH/MASH resolution and fibrosis improvement in F2–F3 disease.	Phase 2b randomized evidence with longer follow-up data.	Not approved; gastrointestinal adverse events and need for phase 3 confirmation remain relevant.	[37] NCT04767529.
**Pirfenidone**	Antifibrotic and anti-inflammatory candidate supported mainly by preclinical MAFLD/NASH data.	Mainly preclinical evidence.	Not approved for MASLD/MASH; lacks large, randomized biopsy-based human trials.	[31,32]
**Statins**	Cardiovascular risk reduction in MASLD/NAFLD patients with dyslipidemia.	Guideline-supported for cardiovascular risk management.	Not a MASH-resolving or antifibrotic therapy.	[3,13]
**Metformin**	Glycemic control and insulin resistance management in patients with type 2 diabetes.	Guideline-supported for diabetes management, not as MASH-specific therapy.	Does not provide meaningful histological benefit in NASH/MASH.	[3,13]

**Table 2:** Nanotechnology-based therapeutic strategies for MASLD: design rationale, experimental evidence, and translational limitations.

Nanotechnology Strategy	Design Purpose	Experimental Model/Clinical Status	Main Limitations	References
**Organelle-targeted nanoparticles**	Intracellular delivery toward dysfunctional organelles involved in oxidative stress, lipid handling, ER stress, autophagy and organelle crosstalk.	Mostly in vitro and preclinical in vivo models.	Requires validation of hepatic accumulation, cellular uptake, endosomal escape, organelle-specific trafficking, toxicology, and scalable manufacturing.	[100,101]
**Mitochondria-targeted nanoparticles, including TPP^+^-functionalized systems**	Mitochondrial delivery to reduce mtROS, preserve mitochondrial function and attenuate oxidative liver injury.	In vitro lipotoxicity models and preclinical diet induced NAFLD/MASLD models.	Mitochondrial accumulation, biodistribution, long-term toxicity, reproducibility and disease-stage specificity require further validation.	[51,101]
**Lysosome/autophagy-targeted and acid-activated nanoparticles**	Restoration of lysosomal acidification, autophagic flux and lipophagy to improve fatty-liver metabolic dysfunction.	Hepatocyte lipotoxicity models and preclinical NAFLD/MASLD models.	Acid-sensitive behavior may vary by hepatic microenvironment and disease stage; translational pharmacokinetics and long-term safety remain unresolved.	[53]
**Plant-derived/exosome-like nanoparticles**	Natural vesicle-mediated delivery of bioactive cargo with antioxidant, anti-inflammatory and hepatoprotective potential.	In vitro hepatic cell models and preclinical NAFLD/MASLD or liver fibrosis models.	Isolation, purification, batch reproducibility, cargo characterization, dose standardization and regulatory classification remain unresolved.	[56,58]
**Polymeric and hybrid nanocarriers—PLGA, PEG, chitosan, PCL and lipid–polymer systems**	Improved solubility, stability, controlled release and liver-cell-directed delivery of therapeutic compounds or nucleic acids.	Mostly in vitro and preclinical in vivo NAFLD/MASH or liver fibrosis models.	Manufacturing complexity, polymer degradation profiles, scalability, reproducibility, long-term safety and interspecies differences limit translation.	[101,102,103]
**Nanoparticle-encapsulated pharmacological or nutraceutical agents**	Enhanced delivery, stability, bioavailability and tissue exposure of therapeutic agents such as curcumin, resveratrol, bilirubin, celastrol, vitamin E or other small molecules.	Predominantly preclinical in vivo models with complementary in vitro validation. Clinical evidence exists mainly for curcumin-based bioavailable/nanoformulated products in NAFLD, including CurcuVail^®^ registered as NCT06256926.	High formulation heterogeneity prevents direct comparison; most systems lack standardized pharmacokinetic, biodistribution and toxicological assessment. Clinical evidence is compound- and formulation-specific.	[101,104] NCT06256926.
**mRNA- and protein-based nanotherapies**	Delivery of therapeutic transcripts or proteins for hepatocyte regeneration, inflammation control, fibrosis modulation or metabolic regulation.	Mainly preclinical liver injury, regeneration, or fibrosis models.	mRNA stability, innate immune activation, repeated dosing, liver-cell specificity, durability of expression and long-term safety remain major barriers.	[105,106]
**siRNA-based nanotherapies and liver-targeted RNAi approaches**	Transient silencing of disease-relevant genes involved in inflammation, lipophagy, autophagy dysfunction and fibrosis.	Preclinical nanoparticle systems exist. Clinically, liver-targeted RNAi/GalNAc-conjugated therapies have entered human testing in NASH/MASH, including ARO-HSD NCT04202354 and ALN-HSD NCT04565717.	Off-target silencing, immune activation, endosomal escape, delivery specificity, durability and long-term safety require further validation. GalNAc-conjugated RNAi therapies should be distinguished from nanoparticle-based siRNA delivery.	[107,108,109]
**CRISPR-based nanotherapies**	Genome editing of disease-related hepatic pathways with potential long-term modulation.	Preclinical and mechanistic models.	Editing safety, off-target mutations, immune responses to editing machinery, irreversibility, delivery specificity and regulatory complexity remain major barriers.	[42,110]
**Other liver-targeted oligonucleotide therapies—antisense/GalNAc-conjugated agents**	Liver-targeted modulation of genes involved in lipid metabolism or MASH susceptibility; included here only as an advanced delivery approach, not as nanoparticle therapy.	Human clinical testing exists. ION224, a ligand-conjugated antisense medicine targeting DGAT2, is registered in MASH/NASH as NCT04932512.	Not nanoparticle-based; should not be grouped mechanistically with nanocarriers. Long-term efficacy, safety and histological benefit remain therapy specific.	[111]

## Data Availability

No new data were created or analyzed in this study.

## Abbreviations

The following abbreviations are used in this manuscript:

8-OHdG	8-hydroxy-2′-deoxyguanosine
acNPs	Acid-activated biodegradable nanoparticles
ALT/AST	Alanine/aspartate aminotransferase
AMLN	Amylin liver NASH diet
APAP	Acetaminophen
Au	Gold (nanoparticles)
BELNs	Blueberry-derived exosome-like nanoparticles
BMI	Body mass index
Cap-LNPs	Capsaicin-lignin nanoparticles
CeO_2_	Cerium oxide
CCl_4_	Carbon tetrachloride
DAMPS	Damage-associated molecular patterns
CDD	Choline-deficient diet
CD-HFD	Choline-deficient high-fat diet
CMNs	Cell-membrane nano-particles
CK	Compound K (
DBZ	Dibenzazepine (PEGylated bilirubin NPs)
DSS	Dextran sulfate sodium
EGF	Epidermal growth factor
Et (chronic)	Chronic ethanol exposure
ER stress	Endoplasmic reticulum stress
EASD	European Association for the Study of Diabetes
EASL	European Association for the Study of the Liver
EASO	European Association for the Study of Obesity
ECM	Extracellular matrix
FA	Fatty acids
FFA	Free fatty acids
FXR	Farnesoid X receptor;
GalNAc	N-acetylgalactosamine
GLP-1	Glucagon-like peptide-1
GSH	Glutathione
HCC	Hepatocellular carcinoma
H-VLNs	Honey vesicle-like nanoparticles
Hep@PGEA	Hepatocyte-targeted poly(glycidyl methacrylate-ethanolamine) vector
HFD	High-fat diet
HFF	High-fat/high-fructose diet
HFHF	High-fat/high-fructose diet
HFCMCD	High-fat/high-cholesterol/methionine- and choline-deficient diet
HGF	Hepatocyte growth factor
HDL	High-density lipoprotein
HMGB1	High mobility group box-1
HSC	Hepatic stellate cell
IL-22	Interleukin-22
IR	Insulin resistance
MASLD	Metabolic dysfunction-associated steatotic liver disease
MASH	Metabolic dysfunction-associated steatohepatitis
mRNA-LNP	Messenger RNA–lipid nanoparticle
MSNs	Mesoporous silica nanoparticles
NASH	Non-alcoholic steatohepatitis
NAFLD	Non-alcoholic fatty liver disease
NanoCys	PEG–poly(cysteine) nanoparticles
NaYF_4_	Sodium yttrium fluoride
NM-FGF19	Non-mitogenic fibroblast growth factor 19
LCS-SeNPs	Chitosan selenium nanoparticles
LDL	Low-density lipoprotein
OA	Oleic acid
PA	Palmitic acid
PI3K/AKT	Phosphoinositide-3-kinase/protein kinase B
PLGA	Poly(lactide-co-glycolide)
PNM	Platelet–neutrophil hybrid membrane
PNPLA3 I148M	Patatin-like phospholipase domain-containing protein 3 (I148M variant)
PPARα	Peroxisome proliferator-activated receptor-α
ROS	Reactive oxygen species
siRNA	Small interfering RNA
SCAR	Steatohepatitis-associated circRNA ATP5B Regulator
SGLT2	Sodium–glucose cotransporter 2
STZ	Streptozotocin
TEF	Naringin treatment in tissue-engineered fatty
TG	Triglycerides
TNF-α	Tumor necrosis factor-alpha
TiO_2_	Titanium dioxide
